# Supplementary material for: Patterns of Intron Gain and Loss in Fungi
Source: PLoS Biol. 2004 Nov 30;2(12):e422. doi: 10.1371/journal.pbio.0020422 (PMC532390; doi:10.1371/journal.pbio.0020422)
Supplement: Table S1 — Also available at http://genes.mit.edu/NielsenEtAl/. (4.3 MB ZIP). [file pbio.0020422.st001.zip › NielsenEtAl/html/1381.html]

AN8834.1.NCU00404.1.MG06322.1.FG05466.1


```
 CLUSTAL W (1.82) Multiple Sequence Alignments - Introns Inserted


Sequence 1: MG06322.1	426 aa
Sequence 2: FG05466.1	415 aa
Sequence 3: NCU00404.1	427 aa
Sequence 4: AN8834.1	435 aa
Alignment Length: 450 aa
Number Identitical Residues: 168 aa
Alignment Score (without introns) 9173


MG06322.1 	MGS-QMGTRAKGASL-SLKTGNKLKRQYLNVRNKKDKDARKRAERFKRRKEENADPTLRQ
NCU00404.1	MPSGNKGKNRSGGSL-SLKTSNKIVRKQLYVKQKKEQGKERHEERHRRRKEEAKDPKLRE
FG05466.1 	MG------FTKPPVQ-ALKSANKLKRKELYVQYKKHQNKEKHEERHNRRREEAKDPELKA
AN8834.1  	MVSKKASARPPSGIIGDFKVRNKQKRQLLHIKRKRAKDAARRAQRFSTKKEEAKNPKLKE
          	* : . .        . :*  **  *: * :: *: :.  :: :*.  ::**  :* *: 

MG06322.1 	ERLAKNKPASQDKKRVWDEGD---DDSLGAIVDLAALKRRRIEEEENA------ALEG--
NCU00404.1	ERLANNQPLTIERKRVWDDVD---DDSLGVAVDVAELKRRRLEKEE--------AEAARL
FG05466.1 	ARLAKRKPATIDSKRVWDDVD---DDSLGVQVDLERLKKRRIEEAEAA------EQAAHE
AN8834.1  	ERLKRNIPLTLERKRVWDDAGSDVEEPLGLSVDVERIKRLKQEEDEELNRPLDSGSEDRH
          	 ** .. * : : *****: .:. ::.**  **:  :*: : *: *  . . .:      

MG06322.1 	AAGLEEQKLDDNDDADSMLDSEEEDDDDDESREE---KLRSQRARRDPSLVPSLAP---S
NCU00404.1	ADGVTEEDEEKDDDADSMLDSDEEGDDDDEDAEERIERLRQERAQRKPSIAP--------
FG05466.1 	AAMRAEEELDEDDDVDSMLASDEEMDEERQAALE---KKRERRAQRDTSVAP--------
AN8834.1  	SEDNGSDEEDDEDDLDSMLASSDEEDEGDEGKES-----KKDSRGRKPSAIPSATERATS
          	:    .:. :.:** **** *.:* *:  :   .     :.    *..*  *: :  :::

MG06322.1 	LAVSTTSTNLDLTPTSLARKFPTLFTEGPHPTPKILVTTSLRATIHREAQIITSLFPNSQ
NCU00404.1	---STTSTNLDMTPDSLTRQFPNLFNEEPPVDPKILVTTHLNGTIHKEAEDIAAIFPNST
FG05466.1 	---STTSTNLDLTPTSLALKFPSLFTDVPAPEPKVLVTTSLNSTLHEQANVLCEFFPNSS
AN8834.1  	PSQSTKSTNLNLAPEALAAKFPSLFSPDSQRPPKILITTSLNSTLHHEAEILTQLFPNSV
          	 : **.****:::* :*: :**.**.  .   **:*:** *..*:*.:*: :  :**** 

MG06322.1 	YVPRSSHRYGHKYSLREICKFSTN~RDFTAVILIREDQKKPTGMTVVHLP-----EGPTF
NCU00404.1	YIRRSAHAYGHKYSVREIAKFAKN1RGYTILMIVGEDLKKPSSLTVIHLNGEDVAPGPSL
FG05466.1 	YVPRSAHRYGHKYSLREICKYATN~REYTAIILLKEDSKKPTGLTVVHLP-----SGPTF
AN8834.1  	YIRRTAHAHAHKFSIREIAKFASN~REYTTLIILQEDSKKPAGLDIVHLP-----KGPMF
          	*: *::* :.**:*:***.*::.* * :* :::: ** ***:.: ::**       ** :

MG06322.1 	HFTINNWIEGSKLPGHGNSQ0GFY~PELLLNRFTTPLGLLTAQLLRRLFPYQPEIAG1RE
NCU00404.1	TYTIRNYLPGKALLGHGNPT~NHY0PELLLNGFKTPLGILAAKSMHTMFPPRPELSG~RQ
FG05466.1 	HFSITNWIEGKKLPGHGNPT~NHY~PELLLNNFKTPLGLLTAKLFMTLFPPKPEFQG~RQ
AN8834.1  	HFSISNWVEGKKIPGHGKPT~EHW~PELILNNFRTPLGLLTAHLFRTLFPPQPDIEG~RQ
          	 ::* *:: *. : ***:.   .: ***:** * ****:*:*: :  :** :*:: * *:

MG06322.1 	VVTIHNQRDYLFVRRHRYVYRERRQTEKTVVDEEGKEIPGFSDIRVGLQEIGPR~FTLKL
NCU00404.1	VVTLHNQRDYIFFRRHRYVFREARTTEKNVVTAEGKEMEGMQGIRAGLQEIGPR1FTLKL
FG05466.1 	VTTLHNQRDYIFVRRHRYVFREKRQTEKSIVGADGKEVKGVEGIRAGLQELGPR~FTLKL
AN8834.1  	VVTLHNQRDYIFVRRHRYVFREKRETEKAVVGADGKEIKGAEGIRTGLQELGPR~FTLKL
          	*.*:******:*.******:** * *** :*  :***: * ..**.****:*** *****

MG06322.1 	RRVDKGIGRAGSEGDDAVQWEWKAGMEKVRTKFNL
NCU00404.1	RRVDKGIGRAGSEGEDALKWEWKAKMEKVRTRFNL
FG05466.1 	RRVDKGIGRAGSDGDDATQWEWKAKMEKDRKRFNL
AN8834.1  	RRVDKGIQRASGQ-----EWEWKGKMEKKRTLFQL
          	******* **..:     :****. *** *. *:*
```
